# Baricitinib Lipid-Based Nanosystems as a Topical Alternative for Atopic Dermatitis Treatment

**DOI:** 10.3390/ph16060894

**Published:** 2023-06-18

**Authors:** Núria Garrós, Paola Bustos-Salgados, Òscar Domènech, María José Rodríguez-Lagunas, Negar Beirampour, Roya Mohammadi-Meyabadi, Mireia Mallandrich, Ana C. Calpena, Helena Colom

**Affiliations:** 1 Departament de Farmàcia i Tecnologia Farmacèutica, i Fisicoquímica, Facultat de Farmàcia i Ciències de l’Alimentació, Universitat de Barcelona (UB), 08028 Barcelona, Spain; ngarroar50@alumnes.ub.edu (N.G.);; 2 Institut de Nanociència i Nanotecnologia, Universitat de Barcelona (UB), 645 Diagonal Avenue, 08028 Barcelona, Spain; 3 Departament de Bioquímica i Fisiologia, Facultat de Farmàcia i Ciències de l’Alimentació, Universitat de Barcelona (UB), Av. Joan XXIII, 08028 Barcelona, Spain

**Keywords:** liposomes, baricitinib, JAK-inhibitor, transepidermal delivery, skin permeation

## Abstract

Atopic dermatitis (AD) is a chronic autoimmune inflammatory skin disorder which causes a significant clinical problem due to its prevalence. The ongoing treatment for AD is aimed at improving the patient’s quality of life. Additionally, glucocorticoids or immunosuppressants are being used in systemic therapy. Baricitinib (BNB) is a reversible Janus-associated kinase (JAK)-inhibitor; JAK is an important kinase involved in different immune responses. We aimed at developing and evaluating new topical liposomal formulations loaded with BNB for the treatment of flare ups. Three liposomal formulations were elaborated using POPC (1-palmitoyl-2-oleoyl-glycero-3-phosphocholine), CHOL (Cholesterol) and CER (Ceramide) in different proportions: (i) POPC, (ii) POPC:CHOL (8:2, mol/mol) and (iii) POPC:CHOL:CER (3.6:2.4:4.0 mol/mol/mol). They were physiochemically characterized over time. In addition, an in vitro release study, ex vivo permeation and retention studies in altered human skin (AHS) were also performed. Histological analysis was used to study the tolerance of the formulations on the skin. Lastly, the HET-CAM test was also performed to evaluate the irritancy capacity of the formulations, and the modified Draize test was performed to evaluate the erythema and edema capacity of the formulations on the altered skin. All liposomes showed good physicochemical properties and were stable for at least one month. POPC:CHOL:CER had the highest flux and permeation, and the retention in the skin was equal to that of POPC:CHOL. The formulations exhibited no harmful or irritating effects, and the histological examination revealed no changes in structure. The three liposomes have shown promising results for the aim of the study.

## 1. Introduction

Lipid-based nanosystems (LBN) are formed through a lipid phase and surfactants [1]. They have demonstrated that they improve the delivery of various active principles to specific skin layers, with stated localization in the upper layers of the skin [2]. There are different kinds of LBN: liposomes, ethosomes, transferosomes, solid lipid nanoparticles, nanostructured lipid carriers, cubosomes, and monoolein aqueous dispersions [3].

The study of liposomes for drug delivery or targeting on specific sites of the body has been going on since 1970. Liposomes possess structural flexibility in terms of their size, composition, surface charge, bilayer fluidity, and their capacity to incorporate virtually any drug irrespective of its solubility, or to display cell-specific ligands on their surface. Consequently, liposomes can be customized in numerous ways so as to create formulations that are ideal for clinical use [4].

**Figure 1:**
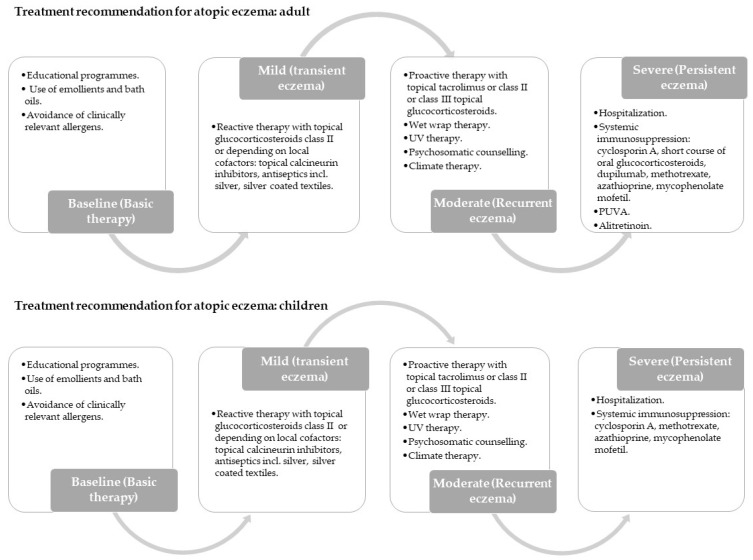
Treatment recommendations for adults and children with atopic eczema according to European guidelines. PUVA = Psoralen and ultraviolet light therapy.

The structural similarity between the lipids composing the nano systems and those composing the skin represents one of the main advantages of LBN, allowing the interaction between the nanosystem matrix and the *stratum corneum* [3]. CHOL, CER and free fatty acids are present in the stratum corneum, the superficial layer of the skin. All of them are involved in different cellular processes at some level, such as transport functions and immune activity [5,6]. Different subclasses of CER exist in the skin, all interacting with the lipids [6]. Patients with AD have a different composition of lipids in their skin, and, in particular, this arises from them having a lower proportion of CER and increased proportions of unsaturated free fatty acids. These differences with normal skin lead to a different organization of the lipids in the skin and thus a different skin structure [7,8,9]. AD affects populations of all ages, particularly children. It is a worldwide chronic autoimmune inflammatory skin disorder highly prevalent in developed countries and it has been increasing over the most recent decades. This disease implies a deterioration of quality of life, worn down by the symptoms and secondary infections [10]. The treatment of the disease will depend on the degree to which these manifestations are present in the patients. It is also very important to be aware of the individual trigger factors so as to avoid them and reduce flare-ups (Figure 1) [11]. The European Academy of Dermatology and Venereology classifies the different treatments, grading them into six signs: erythema, exudation, excoriation, dryness, cracking and lichenification [12]. Additional therapeutic options should be considered in every treatment phase if established therapy is insufficient or in cases of major infections.

Baricitinib (BNB) is a reversible Janus-associated kinase (JAK)-inhibitor. JAK is important because it stimulates the signal transducers and activators of transcription (STAT) that cause different immune responses. These include monocyte activation, antibody secretion, erythropoiesis and acute phase reactant production [13]. Furthermore, BNB has recently been introduced orally in cases of moderate-to-severe AD, showing good results in reducing clinical symptoms and improving the quality of life. The most frequent oral doses used are 2 and 4 mg, resulting in a therapeutic plasma concentration of 0.055 μg/mL [14]. However, it has some side effects such as infectious diseases, cardiovascular events and deep venous thrombosis [15]. Topical drug administration is an alternative to avoid systemic effects. To our knowledge, there are no published studies of BNB for the topical route of administration. The main aim of this work was to develop liposomes loaded with BNB for topical administration using natural lipids as a possible topical alternative to the oral route currently available for patients with AD. To achieve this, the following specific objectives were set: (i) to study the physicochemical characteristics of each liposome differentiated by the formulations of three natural lipids: POPC (1-palmitoyl-2-oleoyl-glycero-3-phosphocholine), CHOL (Cholesterol) and CER (Ceramide) in different proportions; (ii) to investigate their drug release profile, as well as the permeation of BNB through physically altered human skin (AHS), the skin being a model of a compromised permeability barrier, which is a particular feature in AD; and (iii) to study the drug retention capacity on AHS, and the tolerability of the formulations by a modified Draize rabbit test and hen’s egg chorioallantoic membrane test (HET-CAM test). It is worth mentioning that POPC is a natural lipid found in different microorganisms but not in human skin [16], while CHOL and CER are found in the stratum corneum, as previously mentioned.

## 2. Results

### 2.1. Liposomes Physicochemical Characterization

Three liposome formulations were prepared: POPC liposomes, POPC:CHOL liposomes and POPC:CHOL:CER liposomes. The formulations were characterized for pH, particle size and size distribution, encapsulation efficiency and surface charge. This was carried out when the formulations were freshly prepared and when they had been stored at 4 °C.

The pH value for all the liposomes prepared was the same (7.4 ± 0.1), which is adequate for atopic skin treatments [17]. Likewise, the liposomal formulations exhibited comparable efficiency of encapsulation (EE): EE (%) of 6.21 ± 0.55% for POPC; 5.57 ± 0.50% for POPC:CHOL; and 6.21 ± 0.63% for POPC:CHOL:CER.

The results of vesicle size, polydispersity index (PDI) and Zeta potential (ZP) for each liposome are presented in Table 1. All liposomes share similar characteristics, except for liposome POPC:CHOL:CER, which has a higher ZP.

These values were also calculated one month after their elaboration and no-significant change was observed, the maximum being 4%.

### 2.2. In Vitro Drug Release Study

**Figure 2:**
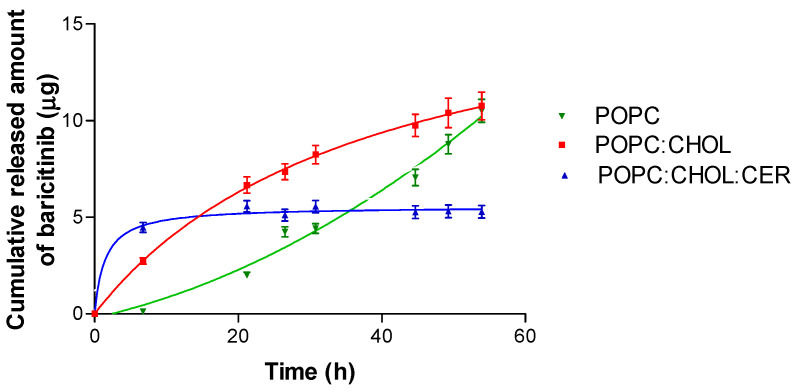
The BNB release profiles from liposomes POPC, POPC:CHOL and POPC:CHOL:CER. BNB cumulative released amount (μg) vs. time (h). The results are presented as mean ± SD (*n* = 5).

The drug release from the liposomes was evaluated by Franz diffusion cells using a dialysis membrane at the cutaneous temperature, 32 °C, yielding the cumulative amount of BNB released as a function of time. The data were then fitted to different kinetic models. The BNB release profiles from the three liposomes varied; Table 2 indicates the corresponding equations. The liposomes POPC:CHOL:CER and POPC:CHOL fitted a hyperbola model, while liposome POPC was in accordance with a polynomial (second order) model. The modeling of the release profile is shown in Figure 2.

The liposome POPC:CHOL:CER releases the drug faster than the other two liposomes; after 8 h, the release of BNB had already reached a plateau, indicating that the maximum amount of the drug that could be released had been reached. Nevertheless, the total amount of drug released is lower than with the other liposomes. In contrast, the liposomes, POPC and POPC:CHOL, have a sustained release of up to 53 h. At this time, those liposomes exhibited a 2-fold drug release compared to liposome POPC:CHOL:CER, as demonstrated in Figure 2.

**Figure 3:**
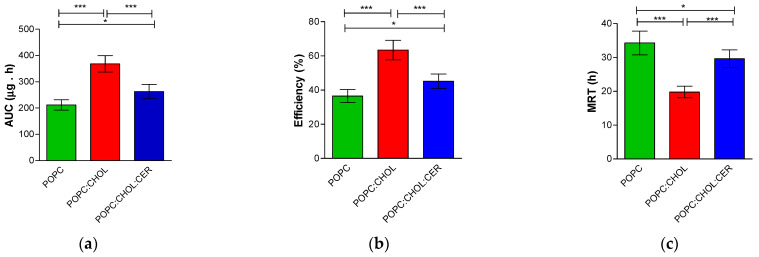
Non-modelistic parameters of: (**a**) area under the curve (AUC: μg/h); (**b**) efficiency (E:%); (**c**) mean release time (MRT: h). A one-way ANOVA test was carried out, followed by Tukey’s multiple comparison test (* *p* < 0.05, *** *p* < 0.0001). The results are presented as the mean ± SD (*n* = 5).

To compare different drug release profiles, non-modelistic parameters such as area under the curve (AUC), release efficiency and mean release time (MRT) were calculated. Figure 3 shows the non-modelistic parameters as well as the statistical parameters test, which confirmed there were differences between the three liposomes. Liposome POPC:CHOL displayed the highest values of AUC and the greatest efficiency percentage, but, in contrast, it has the lowest MRT value.

### 2.3. Ex Vivo Permeation Study

As with the in vitro drug release study, the amount of BNB that was capable of permeating through the human skin was evaluated by Franz cells. Since dermatitis atopic skins present increased permeability relative to healthy skin, the skin was subjected to microneedles to obtain a more permeable skin (hereinafter AHS) and was then mounted on the Franz cells.

**Figure 4:**
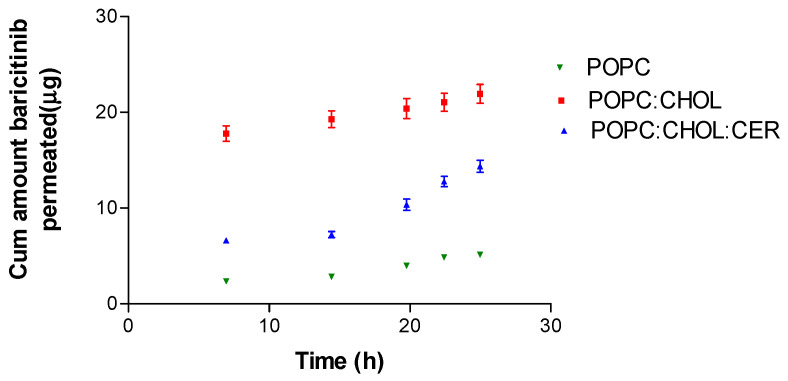
BNB permeation profile: cumulative amount of BNB permeated (μg) vs. time (h). The results are presented as mean ± SD (*n* = 5).

The permeation profile, as the cumulative amount permeated through AHS over 25 h, is outlined in Figure 4. It shows that the liposome POPC:CHOL exhibited the highest permeation, with liposome POPC:CHOL:CER following behind, and liposome POPC showing the lowest permeation of the three liposomes tested. The permeation parameters calculated are presented in Table 3.

**Figure 5:**
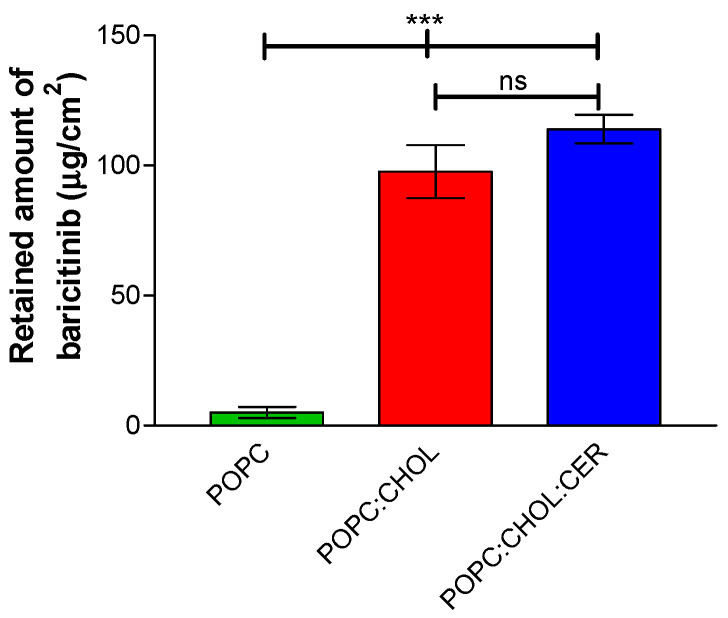
Retained amount (μg/cm^2^) of BNB at 25 h in the AHS. Results are expressed as mean ± SD (*n* = 5). *ANOVA* test analysis of variance, followed by Tukey’s multiple comparison test, were performed. Statistically significant difference: *** = *p* < 0.0001; ns = no statistically significant difference.

Figure 5 illustrates the quantity of BNB retained in the tissues and the corresponding statistical outcomes. Liposome POPC demonstrated significant statistical differences when compared to liposome POPC:CHOL and liposome POPC:CHOL:CER. However, no significant differences were observed between liposome POPC:CHOL and liposome POPC:CHOL:CER.

### 2.4. Tolerance Study and Histological Analysis

#### 2.4.1. In Vivo Tolerance Study

**Figure 6:**
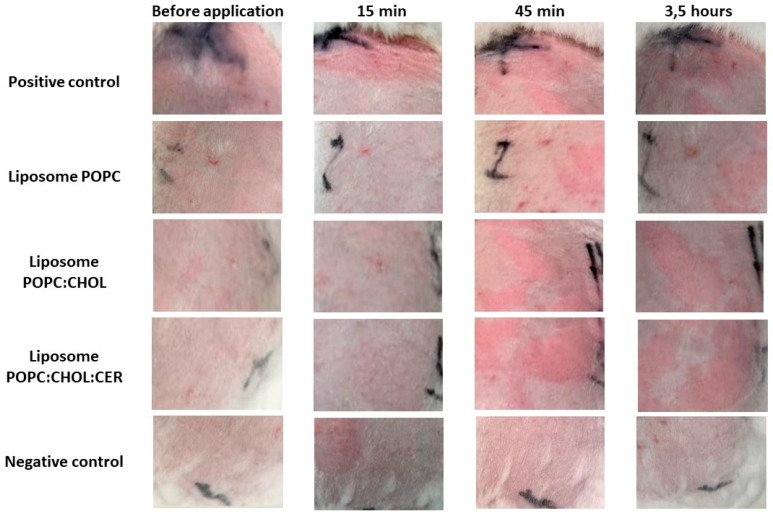
Pictures of 5 groups of rabbits used for the modified Draize test: before the application of xylol and liposomes, at 15 min, 45 min and 3 and a half hours after the respective applications.

The tolerability of the liposomal formulations on altered skin (previously subjected to microneedle punches) was evaluated on New Zealand rabbits. Figure 6 shows the progression of altered rabbit’s skin (ARS) monitored for 3 h 30 min after applying xylol so as to induce erythema and edema. This was intended to stimulate AD flare-ups. All three liposomes appear to delay the onset of edema and erythema caused by xylol. The use of POPC liposomes was found to reduce the damage caused by xylol. The negative control of ARS was not treated with any substance or formulation.

The erythema and edema presented on the rabbits’ backs are classified by the view scoring system (see Table 4).

**Figure 7:**
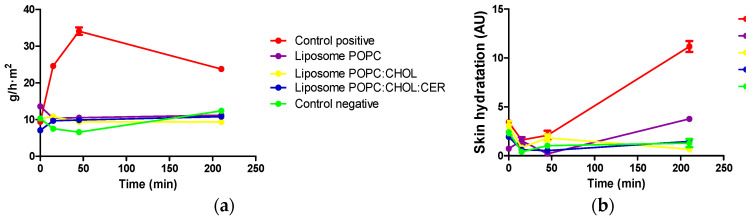
Results of TEWL and skin hydration during 210 min before and after formulations application on rabbits with induced dermatitis by xylol, except for the negative control. (**a**) TEWL; (**b**) Skin hydronation (AU). Results are expressed as mean ± SD (*n* = 5).

The pictures of Figure 6 are supported by the graphs shown in Figure 7, which show the evolution of the transepidermal water loss (TEWL), and the evolution of the skin hydration, of rabbits with altered skin.

#### 2.4.2. Histological Analysis

**Figure 8:**
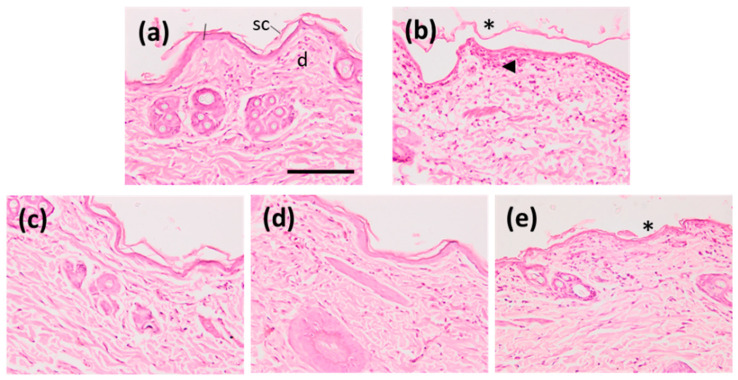
Skin sections colored with eosin and hematoxylin. (**a**) Control negative skin; (**b**) positive control; (**c**) treated with POPC; (**d**) treated with POPC:CHOL; and (**e**) treated with POPC:CHOL:CER. sc = stratum corneum, d = dermis, * shows loss of stratum corneum, ∆ arrowhead indicates leukocyte infiltrate. Magnification = 200×, scale bar = 100 µm.

Once the modified Draize test had been finished, a histological study was conducted to evaluate whether any structural change had occurred to the altered skin due to the application of the formulations. The histology results of the different liposomes, as well as the positive and negative controls, are set out in Figure 8. The skin treated with liposome POPC:CHOL:CER presented stratum corneum loss (Figure 8e) indicating some disruptive effect. This is in contrast to the skins treated with either liposomes POPC or POPC:CHOL, which do not demonstrate any alteration to the skin.

#### 2.4.3. In Vitro Tolerance of the Liposomal Formulations for Periocular Application

AD can also be present on facial skin, with symptoms such as dryness, redness, sensitivity and itching, and facial skin might need the application of soothing formulations. This is why the potential tolerance of the liposomal formulations on the eye, after a periocular application on the eye, was assessed by the in vitro technique HET-CAM of fertilized chicken eggs. Table 5 shows the irritation score (IS) estimated for the liposomal formulations. The three liposomes resulted in non-irritant formulations exhibiting IS values similar to the negative control, whereas the positive control resulted in higher IS values corresponding to a severely irritant substance.

**Figure 9:**
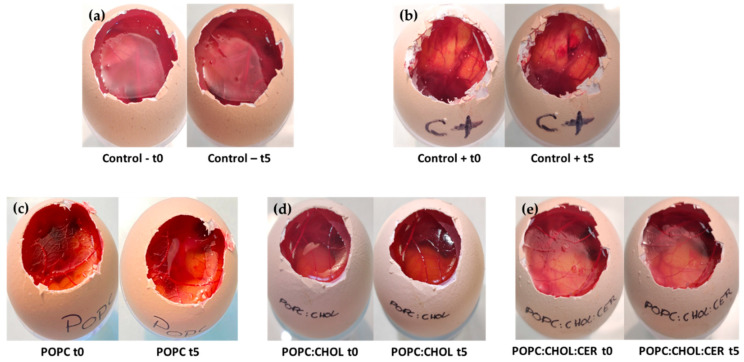
The irritant effect of the formulations was evaluated using the HET-CAM method, with (**a**) a negative control using saline solution, and (**b**) a positive control using 0.1 N sodium hydroxide solution. The other three images show the evolution of the liposomal formulations: (**c**) POPC liposome, (**d**) POPC:CHOL liposome and (**e**) POPC:CHOL:CER liposome.

Figure 9 shows the results from the HET-CAM test. They revealed no irritant effect of any kind; no hemorrhage, coagulation or lysis vessel were observed 5 min after 300 μL of the respective formulations had been applied. This is opposite to the positive control, in which lysis vessel and coagulation appeared (Figure 9b).

## 3. Discussion

All physicochemical characteristics, such as pH, vesicle size and ZP, influence the liposome interaction with the skin [19]. Liposome POPC was the largest vesicle, followed by POPC:CHOL, and the smallest was POPC:CHOL:CER. All of them had a PDI below 0.2, which is indicative of the uniformity of the particle size, and their small size and the low PDI mean the formulations tend to pass more easily through the sterilizing filters, which, in turn, renders them suitable for skins that are grazed or scratched and could become infected. Instead, the ZP helps to predict the stability of particles. A high ZP indicates a stable formulation, so the liposome POPC:CHOL:CER is the most stable of the tested liposomes [20]. Differences in the lipid used for each liposome elaboration may lead to different release profiles described by different kinetic models [21]. In our work, the two liposomes containing CHOL displayed the same kinetic model suggesting that the presence of CHOL in the liposome strongly impacts on their drug release characteristics. The fastest BNB release over a short period of time was presented only by liposome POPC:CHOL:CER but not by liposome POPC:CHOL (Figure 2). Although liposomes POPC and POPC:CHOL exhibited similar BNB released at 54 h, POPC:CHOL showed a higher AUC than POPC:CHOL:CER, indicating a superior performance by POPC:CHOL. The mathematical modelling in the release studies is an excellent tool to evaluate differences between formulations [22,23,24]. Furthermore, the non-modelistic parameters are useful to compare formulations which exhibit different kinetic models. In this work, the non-modelistic parameters showed a relation between the amount of BNB that is released from the formulation and the release efficiency, with the cumulative permeated amount throughout the 25 h period being the liposome POPC:CHOL, the formulation with the highest values. Baricitnib is a molecule with poor water solubility under physiological conditions, as observed from its structure. It does not present charged structures at pH 7.40, suggesting that it could be found deep within the lipid bilayer. Moreover, it presents several groups capable of stabilizing the molecule in hydrophobic regions through hydrogen bonds. These facts point to baricitinib incorporating into the liposome lipid membrane, remaining there and not being released to permeate through the skin. To overcome this problem, liposomes containing baricitinib were supplemented with 5% Transcutol. The structure of Transcutol allows it to be incorporated in parallel to hydrocarbon chains with its OH group near the headgroup of the phospholipids, distorting the phospholipid bilayer. Then, when liposomes containing baricitinib interact with the skin surface, baricitinib can easily be released from the liposome, and Transcutol molecules can increase the water solubility of baricitinib and facilitate its distribution towards the surface of the skin.

Different authors have demonstrated that, if the lipids are like those in the stratum corneum, more drugs will penetrate the skin [19]. It is worth mentioning that human skin contains CHOL and CER and disturbances in their levels cause disruption of the skin barrier function. Yet, harmonizing the CHOL and CER levels in the skin restores its barrier function [25]. Sinico and Fadda explained that a greater permeation and release could be produced when the liposomes are composed of lipids that are more similar to those of the skin [26]. This would explain the higher permeation of POPC:CHOL and POPC:CHOL:CER compared with liposome POPC without CHOL. The liposome POPC:CHOL:CER triplicated the results values of flux, permeability coefficient and theoretical plasma concentration in human steady state compared with the other studied liposomes (POPC and POPC:CHOL, see Table 3). Therefore, CER could be acting like a permeation enhancer. CERs have been considered the principal factor in the control of the skin barrier as the enhancer effect of CER analogues has been demonstrated in diverse studies [27]. Since the predicted plasma concentration at steady state did not reach therapeutic levels, it is likely that BNB only exerts a local effect on the skin [14].

Retention of BNB in the skin was not statistically different between POPC:CHOL and POPC:CHOL:CER, so both liposomes would be a good option in order to achieve a local effect of BNB because both liposomes reached values a hundred times higher than liposome POPC (Figure 5). Liposomes with CHOL and CER—which are the lipids found in the skin—were able to diffuse through the skin, thus creating a reservoir effect in the BNB. This achieved concentrations inside the skin higher than those obtained in the blood in oral therapy for AD treatment [28], considering the human skin density to be 1 g/mL [29].

The HET-CAM test showed no irritation effect caused by any of the three liposomes and, in reference to the modified Draize test, we observed a good Draize score for erythema and edema (Table 4). Liposome POPC effectively counteracted the impact of xylol on the skin and neither erythema nor edema were observed over the 3 h of the study. Xylol caused both reactions, erythema and edema, which were observed from the first assessment (after 15 min) and up to the end of the tolerance study, which was after 3 h. Liposomes POPC:CHOL and POP:CHOL:CER also effectively counteracted the edema caused by xylol. However, after 45 min of application, a slight erythema appeared in the areas where liposomes POPC:CHOL and POPC:CHOL:CER were tested. Nevertheless, this effect was more than 2-fold and 3-fold lower than the erythema caused by xylol. TEWL values were constant for all the liposomes with similar values to the negative control (within the range of 8–15 g/h·m^2^, which means a healthy value for rabbits), whereas the positive control showed a significant increase. The results are in accordance with those obtained in control rabbits, as observed by Babu M. Medi and Angela Anigbogu [30,31]. The skin hydration values were constant and followed the trend of the negative control, while the positive control showed a steady increase during the tolerance study. Finally, the histological study demonstrated that liposome POPC and POPC:CHOL avoided any damage to the tissue structures. Based on these promising results, further biochemical studies should be carried out to test the efficacy of these liposomal formulations on animal models of the disease, including mutant animals, before proceeding to clinical trials.

## 4. Materials and Methods

### 4.1. Materials

BNB, Ammonium Formate and POPC were purchased from Sigma–Aldrich (Madrid, Spain). Transcutol^®^ P [Diethylene glycol monoethyl ether] was bought at Gattefossé (Barcelona, Spain). Fisher Chemical (Loughborough, UK) supplied the Acetonitrile. Finally, CHOL (ovine wool > 98%) and CER (bovine spinal cord ≥ 98%) were acquired at Avanti Polar Lipid Inc. (Alabaster, AL, USA). A microneedle roller (Currentbody, Barcelona, Spain) contains 540 titanium needles that are 0.25 mm long, corresponding to 72.3 microneedles/cm^2^.

**Figure 10:**
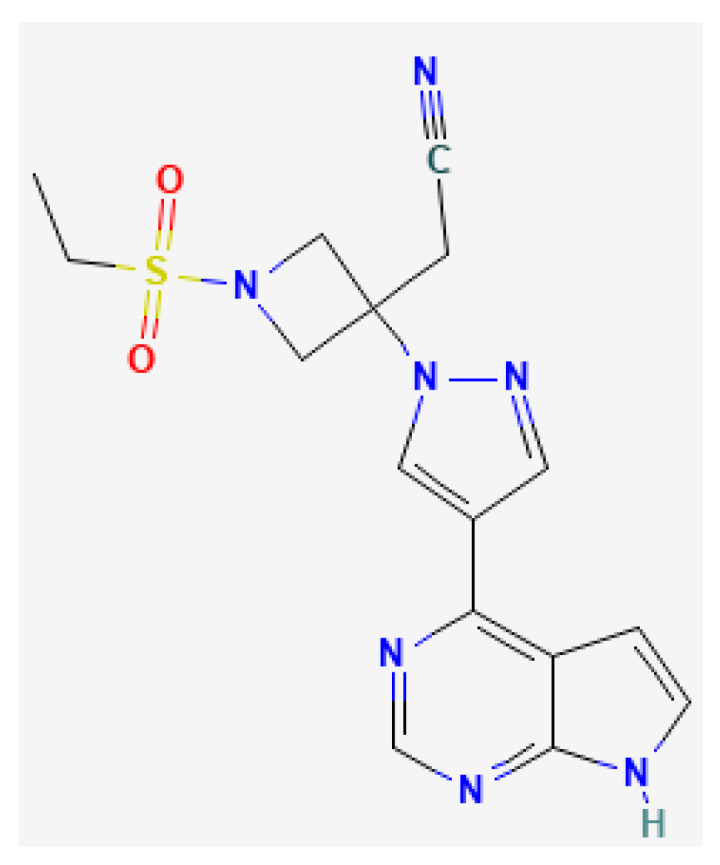
Chemical structure of BNB.

BNB (C16H17N7O2S) is a pyrrolopyrimidine, the chemical structure of which is shown in the figure below, Figure 10, which has been obtained from PubChem, an open chemistry database at the National Institute of Health (NIH).

### 4.2. Biological Materials

The abdominal human skin (protocol code 93-01162 02/18 approved on 17 January 2020 by the 99 Bioethics Committee of SCIAS Hospital de Barcelona), was dermatomed to 400 µm thickness (Aesculap GA 630, Aesculap, Tuttligen, Germany). All the skin was subjected to a physical alteration with microneedles in order to simulate an AD skin by piercing the epidermis. Since the needles do not come into contact with the nerves or blood vessels, this technique is painless when performed on live animals.

### 4.3. Methods

#### 4.3.1. Preparation of the Liposomes

Three different liposome formulations were prepared: (i) pure POPC liposomes, (ii) POPC:CHOL (0.8:0.2, mol/mol) liposomes and (iii) POPC:CHOL:CER (0.36:0.24:0.40, mol/mol/mol) liposomes.

All liposomes samples were elaborated in accordance with the methods published in other articles [32]. In short, to elaborate each liposome sample, we added 500 mg of BNB in a round bottom flask to the corresponding chloroform–methanol (2:1, *v*/*v*) lipid solutions to achieve the desired molar lipid concentration for each composition. Then, the mixture was sonicated for 10–15 s to make sure all BNB was dissolved. Following the removal of the solvent using a rotary evaporator, the thin lipid film was kept in a high vacuum overnight in the absence of light to ensure the absence of organic solvent traces. The thin films were rehydrated using a solution containing 10 mM TRIS·HCl ([tris(hydroxymethyl)aminomethane] 150 mM NaCl), and 5% (*v*/*v*) of Transcutol^®^ P pH 7.40. Vigorous vortexing was performed for 5 cycles, at a temperature above that of the lipid mixture’s transition, so as to obtain large multilamellar vesicles. The liposome size was homogenized using an ultrasound bath with temperature control for 15 min. Finally, the liposomes were passed through a Sephadex^®^ G50 column mounted in a 5 mL syringe and centrifuged at 1000 rpm for 10 s using a Rotanta 460R centrifuge (Andreas Hettich GmbH & Co. KG, DE, Tuttlingen, Germany) to eliminate non-encapsulated BNB.

#### 4.3.2. Liposomes’ Physicochemical Characterization

The physicochemical properties of the liposomes were analyzed using various parameters, including pH, vesicle size, polydispersity index, ZP and encapsulation efficiency. pH measurement was carried out, using a pH-meter micro pH 2001 (Crison Instruments SA, Alella, Spain), in triplicate. The Zetasizer Nano S (Malvern Instrument, Malvern, UK) was used to determine the liposomes size, PDI and ZP. All the physicochemical characteristics were determined in triplicate [33]. All the measures were analyzed by one-way ANOVA test followed by a Tukey’s multiple comparison test. These measurements were repeated on samples stored for up to 1 month at 4 °C to assess the stability of the liposomes.

To study the EE percentage (EE%), the liposomes were broken down with Transcutol^®^ P: Triton at 10% (8:1), so that they released the BNB, and quantified using HPLC (see Section 4.3.6). The comparison between the initial amount and the extracted amount gave the *EE*% (Equation (1)) [32]:

(1) $$EE\%=\frac{Q_{f}}{Q_{0}}\times 100$$

where *Q_f_* is the total mass of BNB retained inside the liposomes and *Q*_0_ is the total mass of BNB initially used to prepare the liposomes. Both quantities are expressed in mg.

#### 4.3.3. In Vitro Drug Release Study

In order to investigate the release of the drug, we employed a dialysis membrane that had a molecular cut-off weight of 14,000 Da (Sigma–Aldrich, Madrid, Spain) in Franz-type diffusion cells. These cells had a diffusion area of 0.64 cm^2^ and a receptor chamber of 4.9 mL (Crown Glass Company, Inc., Jersey City, NJ, USA) [34,35]. Prior to use, the membrane was hydrated in methanol:water (1:1) for 24 h and then washed before being set in the Franz diffusion cells. The receptor medium used was Transcutol^®^ P which was stirred at 500 r.p.m. to keep sink conditions. The experiment was conducted at 32 °C by means of a thermostatic water bath. An amount of 500 μL of formulation was added to the donor compartment; five replicates per each liposome were included. During the experiment, samples of 200 μL were collected at specific time intervals and, in order to maintain a constant volume in the cells, Transcutol^®^ P was added after each sample collection. The collected samples were analyzed by a validated HPLC-fluorescence method, as described in Section 4.3.6. The cumulative amounts of BNB released from each liposomal formulation were plotted over time, and the data were analyzed by different kinetic models to describe the drug release profile. The determination coefficient (r^2^) was used to assess the goodness of fit [36].

Moreover, some non-modelestic parameters were calculated to compare different release profiles. These estimated parameters were the MRT of BNB from the formulation, the AUC representing the amount of BNB that was released from the formulation [37] and the efficiency (E) as the percentage of BNB released from the initial formulation.

#### 4.3.4. Ex Vivo Permeation Study

The permeation studies were conducted using Franz diffusion cells with a surface area measuring 0.64 cm^2^ and a receptor chamber of 4.9 mL. The altered abdominal human skin was placed between the donor and the receptor compartments [34,35]. The receptor medium was Transcutol^®^ P. Amounts of 500 μL of each liposome was put on the donor compartment. Samples of 200 μL were taken over 25 h and replaced by Transctuol^®^ P after every sampling time. We analyzed the samples using the validated HPLC-fluorescence method (see Section 4.3.6).

Three permeation parameters of each liposome were calculated: flux (*Jss*, µg/h), permeability coefficient (*Kp*, cm/h) and theoretical predicted plasma concentration in human steady state applied to a 10 cm^2^ surface (*Css*, ng/mL). Flux is the slope calculated with the aligned points in the permeation profile [35]. The permeability coefficient was obtained by the following equation:

(2) $$Kp=\frac{Jss}{C_{0}\cdot A}$$

where *C*_0_ (μg/mL) is the initial concentration of BNB in the liposome and ***A*** is the surface of the diffusion area (cm^2^) [34].

*Css* was calculated by the equation detailed below:

(3) $$C_{ss}=\frac{Jss\cdot TSA}{Clp\cdot A}$$

where *Jss* (μg/h) is the flux, *TSA* (cm^2^) is the theoretical surface area of application, *Clp* (ml/min) is the human plasma clearance of BNB and *A* (cm^2^) is the diffusion area of the Franz cells [34]. The area of application considered was 10 cm^2^.

After completing the permeation study, we removed all tissues from the diffusion cells and washed away any liposomes remaining on the surface with distilled water. Then, we extracted the BNB that was retained in the permeation area of the tissue, cutting it, weighing it and immersing it in 1 mL of Transcutol^®^ P. The sample was then sonicated for 10 min using an ultrasonic water bath [33]. The final step was to analyze the Transcutol^®^ P with the extracted BNB using HPLC. We calculated the retained BNB in the tissues (*Q_ret_*) using Equation (4) and the results were stated, normalized by the weight of the tissue and the diffusion area (0.64 cm^2^) and multiplied by the recuperation of the drug:

(4) $$Q_{ret}=\frac{Q_{ext}}{W\times A}\times \frac{100}{R}$$

In this equation, *Q_ext_* represents the quantity of drug extracted from the tissue and is measured in µg, *W* denotes the weight of the tissue in grams, while *A* is the diffusion area in cm^2^. Finally, *R* represents the proportion of BNB that is recovered in each tissue [35].

#### 4.3.5. Baricitinib Determination by HPLC

The measurement of BNB in each sample was carried out using high-performance liquid chromatography (HPLC) equipped with a fluorescence detector. The HPLC system consisted of a Chromatograph Waters Alliance 2695 and a Fluorescence Jasco FP-1520 detector that operated at an excitation wavelength of 310 nm and an emission wavelength of 390 nm. The chromatographic column was a Symmetry C18 (4.6 × 75 mm, 3.5 µm) and the mobile phase was Ammonium Formate 10 mM pH 7.4 (75:25 *v*/*v*); the flux was 1 mL/min and the volume of injection was 10 μL. The validated range for the quantification of BNB was from 0.03 to 1 µg/mL.

#### 4.3.6. Tolerance Study and Histological Analysis

Liposomes were also evaluated by a modified Draize skin test to study the effect on induced erythema and edema with xylol to simulate atopic skin. We used non-anesthetized New Zealand healthy rabbits (Harlan, Barcelona, Spain). They were cared for in accordance with the standard conditions, receiving food and water ad libitum. The objective was to detect the possible signs of damage on altered skin as indicated by the level of erythema and edema [38,39]. The studies were conducted under a protocol in accordance with the Mexican Official Norm for Animal Care and Handling (NOM-062-ZOO-1999).

To obtain an ARS, we used microneedles so as to compromise the skin barrier function on the rabbits’ backs. This was carried out one day after having measured their basal values and having shaved them. The animals were divided into different groups: the negative control group which only underwent microneedles; the positive control group which, in addition to the microneedles, received xylol to induce skin irritation; 3 other groups which received microneedles, plus xylol, plus one of the liposomes. Next, we applied 0.5 mL of xylol to all the rabbits minus the negative control (which also did not have liposomes). The positive control received microneedles plus xylol.

The responses were recorded at 15 min, 45 min and 3 h 30 min. At the same points in time, the transepidermal water loss (TEWL) and skin hydration were measured. For the positive control, 0.5 mL of xylol was applied and evaluated at the same times as the liposomes. A visual scoring system was used to evaluate inflammation; this system classifies from 1 to 4 according to whether the erythema and edema were practically imperceptible or had reached a large area of exposure [40]: 0 denotes no erythema and no edema, 1 denotes a little sign of erythema or edema, 2 denotes explicit erythema or slight edema, 3 denotes a moderate to severe erythema or edema and 4 refers to serious erythema (beet redness) through to the first signs of depth injuries or severe edema [41].

Xylol was used as an irritant component to induce allergic contact dermatitis (ACD), characterized by manifestations such as skin redness, swelling, warmth and itching and accompanied by dryness and flaking. Other articles, like those of Patricia L Nadworny et al. [42] or Liu Tang et al. [43], used dinitrochlorobenzene, or Paul L. Stanley et al. [44], who used 12-O-tetradecanoylphorbol-13-acetate (TPA).

TEWL is the amount of water that can diffuse through the skin stratum corneum per unit of time. The appropriate structure of intracellular lipids in the stratum corneum is one of the key factors in retaining transdermal water [45,46]. TEWL was measured by Tewameter (TEWL-Dermalab, Agaram Industries, India). An increment of this value means there is damage on the skin barrier [47]. The skin hydration was measured by a Corneometer (EnviroDerm, Ireland), which can quantify the amount of water within the corneum.

After monitoring the mentioned parameters for 3 h, the treated animal parts were histologically evaluated to assess the effect of the liposomes. To ensure this, the animals were euthanized with xylazine (Rompun^®^ 20 mg/mL, Bayer Hispania, Sant Joan Despí, Spain) and ketamine (Imalgene^®^ 100 mg/mL, Boehringer Ingelheim Animal Health España, Sant Cugat del Vallès, Spain). The mixture was injected via the right ear vein at 4 mg/kg [48]. After clinical death, the back skin was immediately excised, rinsed with PBS pH 7.4, and set overnight at room temperature in 4% buffered formaldehyde and subsequently embedded in paraffin wax. Transverse sections measuring 5 µm were stained with hematoxylin and eosin and then examined using a light microscope (Olympus BX41 and camera Olympus XC50) on blinded-coded samples to assess the histological structure [49].

Finally, a HET-CAM test was conducted to evaluate the potential risk of causing irritation to the eye after a periocular application. The HET-CAM test assesses the potential toxicity of formulations when applied to the CAM of a 10-day embryonated hen’s egg (supplied by the G.A.L.L.S.A. farm, Tarragona, Spain). The CAM was observed for five minutes following application, with any reactions, such as bleeding (hemorrhage), blood vessel disintegration (coagulation) and protein denaturation (intra- and extra-vascular vessel lysis coagulation), being noted [18]. To this end, we applied 300μL of liposomes to the CAM and we waited 5 min to see if there had been any reaction working with the INVITTOX protocol [50]. The positive control used was a 0.1 N solution of NaOH, while the negative control was a solution containing 0.9% NaCl [50]. The IS was calculated, as described by Garrós et al., including 3 replicates per formulation [49].

## 5. Conclusions

Three liposomal formulations containing BNB, a Janus kinase inhibitor, were developed for the topical treatment of complement flare-ups in AD. The liposomes were prepared using three different lipids: POPC, CHOL and CER. The POPC:CHOL:CER formulation showed higher flux and permeation compared to the other formulations. However, there were no statistically significant differences in the retention concentration in the skin between POPC:CHOL and POPC:CHOL:CER. These formulations also showed that they retained higher amounts of BNB than liposome POPC; this may be due to them having a longer effect. However, further biomedical investigations should be carried out. The formulations did not demonstrate any irritant effect in the HET-CAM test, and the liposomes POPC and POPC:CHOL did not cause any structural alteration according to the histological analysis.

## Figures and Tables

**Table 1:** Liposomes composition: hydrodynamic diameter, PDI and ZP; and physical stability for 1 month. The results are presented as mean ± SD (*n* = 3).

Composition	Hydrodynamic Diameter (nm)		PDI		Zeta Potential (mV)	
	1 Day	1 Month	1 Day	1 Month	1 Day	1 Month
POPC	86.0 ± 1.4	86.9 ± 1.1	0.120	0.119	−14.2 ± 0.3	−13.9 ± 0.8
POPC:CHOL (8:2, mol/mol)	53.5 ± 0.9	55.4 ± 1.3	0.174	0.172	−13.2 ± 1.3	−13.5 ± 0.9
POPC:CHOL:CER (3.6:2.4:4.0 mol/mol/mol)	64.1 ± 0.3	65.0 ± 0.7	0.120	0.117	−18.3 ± 1.9	−18.1 ± 1.3

A one-way ANOVA test was carried out and then followed by Tukey’s multiple comparison test. All three liposome sizes are statistically different from each other (*p* < 0.0001). Similarly, the ZP of POPC:CHOL:CER is different from the other two liposomes (*p* < 0.05). Liposome POPC and POPC:CHOL do not have a significantly different ZP compared to each other.

**Table 2:** Liposome formulations’ best-fit values in the kinetic release modeling.

Liposome	Equation	Best-Fit Values According to the Equation				
		A	B	C	B_max_ (μg)	*K_d_* (h)
POPC	Polynominal: Second Order y = A + Bx + Cx^2^	0.21	0.08	0.002	-	-
POPC:CHOL	One site binding (hyperbola) y = B_max_x/(*K_d_* + x)	-	-	-	18.37	38.34
POPC:CHOL:CER	One site binding (hyperbola) y = B_max_x/(*K_d_* + x)	-	-	-	5.55	1.45

A, B and C = parabola coefficients. Hyperbola parameters B_max_ = maximum amount of BNB that can be released; and Kd = drug release constant.

**Table 3:** Permeation parameters of three liposomes: cumulative amount of BNB permeated at 25 h (*AP_25h_*, µg), flux (*Jss*, µg/h), permeability coefficient (*Kp*, cm/h) and predicted steady-state plasma concentration in human steady state on a 10 cm^2^ surface application (*Css*, ng/mL).

	*AP_25h_ (*µg)	*Jss* (µg/h)	*Kp* (10^−4^ cm/h)	*Css* (ng/mL)
POPC	5.13 ± 0.52	0.22 ± 0.03	1.14 ± 0.16	0.36 ± 0.05
POPC:CHOL	21.93 ± 2.20	0.29 ± 0.03	1.52 ± 0.16	0.48 ± 0.05
POPC:CHOL:CER	14.36 ± 1.40	0.77 ± 0.07	4.01 ± 0.37	1.28 ± 0.12

ANOVA test analysis of variance, followed by Tukey’s multiple comparison test, were performed, and all were statistically different from each other (*p* < 0.001). Results are expressed by mean ± SD (*n* = 5).

**Table 4:** A modified Draize score was employed to evaluate erythema and edema resulting from xylol exposure, both with and without subsequent application of the liposomes (mean ± SD, *n* = 5).

Chemicals	Before Induced Er and Ed		15 min		45 min		3 h	
	Erythema	Edema	Erythema	Edema	Erythema	Edema	Erythema	Edema
Negative control	0.00 ± 0.00	0.00 ± 0.00	0.00 ± 0.00	0.00 ± 0.00	0.00 ± 0.00	0.00 ± 0.00	0.00 ± 0.00	0.00 ± 0.00
Liposome POPC	0.00 ± 0.00	0.00 ± 0.00	0.00 ± 0.00	0.00 ± 0.00	0.00 ± 0.00	0.00 ± 0.00	0.00 ± 0.00	0.00 ± 0.00
Liposome POPC:CHOL	0.00 ± 0.00	0.00 ± 0.00	0.00 ± 0.00	0.00 ± 0.00	1.00 ± 0.53	0.00 ± 0.00	0.65 ± 0.40	0.00 ± 0.00
Liposome POPC:CHOL:CER	0.00 ± 0.00	0.00 ± 0.00	0.00 ± 0.00	0.00 ± 0.00	1.45 ± 0.42	0.00 ± 0.00	0.37 ± 0.03	0.00 ± 0.00
Positive control (xylol)	0.00 ± 0.00	0.00 ± 0.00	3.54 ± 0.20	1.62 ± 0.41	3.50 ± 0.40	1.84 ± 0.13	2.40 ± 0.30	0.50 ± 0.02

Er = erythema; Ed = Edema.

**Table 5:** Irritation score calculated for the liposomal formulations (mean ± SD of *n* = 3). The scores obtained for the positive and negative controls are also reported.

	Formulation				
	Negative control	Positive control (0.1 N NaOH)	POPC	POPC:CHOL	POPC:CHOL:CER
Irritation score (IS)	0.07 ± 0.00	16.10 ± 0.08	0.07 ± 0.00	0.07 ± 0.00	0.07 ± 0.00

IS ≤ 0.9, non-irritating/slightly irritating; 0.9 < IS ≤ 4.9, moderately irritating; 4.9 < IS ≤ 8.9, irritating; and 8.9 < IS ≤ 21, severely irritating [18].

## Data Availability

The data presented in this study are available on request from the corresponding author.
